# Ubiquitin-specific protease 3 attenuates interleukin-1β-mediated chondrocyte senescence by deacetylating forkhead box O-3 via sirtuin-3

**DOI:** 10.1080/21655979.2021.2012552

**Published:** 2022-01-16

**Authors:** Qi Zhou, Wei Wang, Jun Wu, Shang Qiu, Shuai Yuan, Pei-Liang Fu, Qi-Rong Qian, Yao-Zeng Xu

**Affiliations:** a Department of Orthopedics, The First Affiliated Hospital of Soochow University, Suzhou PR China; b Department of Orthopedics, Changzheng Hospital, Naval Medical University, Shanghai PR China; c Department of Orthopedics, The Affiliated Hospital of Xuzhou Medical University, Xuzhou Jiangsu, PR China

**Keywords:** Osteoarthritis, senescence, ubiquitination, deacetylation, FOXO3, SIRT3

## Abstract

Osteoarthritis (OA) affects approximately 12% of the aging Western population. The sirtuin/forkhead box O (SIRT/FOXO) signaling pathway plays essential roles in various biological processes. Despite it has been demonstrated that ubiquitin-specific protease 3 (USP3) inhibits chondrocyte apoptosis induced by interleukin (IL)-1β, the role of USP3/SIRT3/FOXO3 in the senescence of chondrocytes in OA is unclear. This study initially isolated articular chondrocytes and investigated the role of USP3 in IL-1β-induced senescence of chondrocytes. After USP3 was overexpressed or silenced by lentivirus, expressions of genes and proteins were detected using quantitative polymerase chain reaction and immunoblotting, respectively. Cell cycle analysis was performed using flow cytometry. Reactive oxygen species (ROS) levels and senescence were analyzed. Then, SIRT3 was inhibited or overexpressed to explore the underlying mechanism. We found that overexpression of USP3 hindered IL-1β-mediated cell cycle arrest, ROS generation, and chondrocyte senescence. The inhibition of SIRT3 blocked the protective effect of USP3 on cell senescence, whereas the overexpression of SIRT3 abolished USP3-silencing-induced cell senescence. Furthermore, SIRT3 attenuated cell senescence, probably by deacetylating FOXO3. USP3 upregulated SIRT3 to deacetylate FOXO3 and attenuated IL-1β-induced chondrocyte senescence. This study demonstrated that USP3 probably attenuated IL-1β-mediated chondrocyte senescence by deacetylating FOXO3 via SIRT3.

## Introduction

Osteoarthritis (OA), a non-inflammatory disease resulting in articular cartilage deterioration, is characterized by joint pain, loss of mobility, joint deformity, and dysfunction[1]. OA affects approximately 12% of the aging population[2]. The most common pathological OA change is the degeneration or loss of articular cartilage, due to the aging process in chondrocytes. The matrix interleukin 1 beta (IL-1β) has been implicated as a key factor regulating OA [3–6]. Some research has revealed that IL-1β contributes to OA development by decreasing the synthesis of the key structural proteins, stimulating the synthesis of other cytokines like tumor necrosis factor-alpha (TNF-α) and IL-6, and promoting the secretion of tissue inflammatory effectors cyclooxygenase-2 and phospholipase A2 [7–9]. Furthermore, studies have shown that chondrocytes subjected to IL-1β treatment tend to age more rapidly and induce apoptosis [10,11].

Oxidative stress is a situation in which cellular levels of reactive oxygen species (ROS) exceed its antioxidant capacities, and accumulation of damage inflicted by ROS is currently widely accepted as a cause of aging [12,13]. High concentrations of ROS directly induce deoxyribonucleic acid (DNA) damage, contributing to aging[14]. Dysregulation of p16 and p21 gene expression is implicated in oxidative stress-induced cell senescence and aging [15,16]. The activation of p21 gene leads to cell cycle arrest [17,18].

The sirtuin (SIRT) family plays essential roles in various biological processes including gene silencing, anti-stress responses, apoptosis, aging, and inflammation [19–21]. A study has demonstrated that SIRT1 depletion can promote senescence of endothelial cells and podocytes[22], whereas the upregulation of SIRT1 can deacetylate p53 and then inhibit the senescence of endplate chondrocytes[23]. Moreover, overexpression of SIRT6 is demonstrated to inhibit chondrocyte aging and suppress nuclear factor kappa B (NF-κB)-mediated inflammation in OA[24]. The overexpression of SIRT3 suppresses hyperglycemia-induced senescence of human diploid fibroblasts by deacetylating FOXO1 and increasing catalase and manganese superoxide dismutase (MnSOD) in WI-38 cell and is implicated as a potential target to treat diseases of senescence[25]. In addition, studies have shown that SIRT3 can protect the mitochondria against oxidative damage by regulating FOXO3 deacetylation, and the mitochondrial oxidative stress and mtDNA damage in osteoblasts can be induced by inhibiting the SIRT1-FoxO3a-MnSOD pathway in osteoporosis [26–28]. Despite the importance of SIRT3/FOXO3 signaling has been well-recognized, its role in chondrocyte aging needs to be elucidated.

Our previous studies have shown that USP3 suppresses chondrocyte apoptosis induced by IL-1β through the de-ubiquitination of TNF receptor-associated factor 6 (TRAF6)[29]. Since the pathological OA change is correlated to the degeneration or loss of articular cartilage, the upregulation of SIRT1 can inhibit the senescence of endplate chondrocytes. In addition, SIRT3 protects the mitochondria against oxidative damage. It is hypothesized that the USP3 can inhibit the IL-1β induced chondrocytes senescence via SIRT3. However, the function of USP3 in the senescence of chondrocytes in IL-1β-induced OA is to be fully explored. Therefore, this study aims at investigating the potential roles of USP3/SIRT3/FOXO3 in IL-1β-induced chondrocyte senescence.

## Materials and methods

### Cell isolation and culture

Knee joints of Sprague-Dawley (SD) rats were prepared and articular chondrocytes were subsequently isolated and identified as previously described[29]. The collected cells were cultured in DMEM/F12 (Sigma-Aldrich, China) with 10% fetal bovine serum (Invitrogen, China) and 1× Pen–Strep (Beyotime, China). The experiments of the current research gained approval from the Ethics Committee of the First Affiliated Hospital of Soochow University.

### Plasmid construction and cell transfection

SIRT3 coding sequence was synthesized employing primers containing EcoR I and BanH I restriction enzyme cutting sites and subsequently incorporated to pLVX-Puro to improve the expression of SIRT3, including SIRT3-F: 5′-CGGAATTCATGGTGGGGGCTGGCATC-3′, and SIRT3-R: 5′-CGGGATCCTTATCCGTCCTGTCCATCCAG-3′. Overexpression (oeUSP3) or silence (siUSP3) of USP3 was performed as previously described[29].

### Quantitative polymerase chain reaction (qPCR)

Ribonucleic acid (RNA) samples were obtained from treated cells by supplying TRIzol reagent and subsequent reverse transcription was performed to complementary DNA (cDNA) using Superscript II (Invitrogen, China). cDNA was amplified based on the following primers employing SYBR Green Master Mix (Thermo Fisher Scientific, China) (Table 1). Relative fold changes were quantified using the 2^−ΔΔCt^ formula[30].

**Table 1.** PCR primer sequences

Gene	Sequence (5′–3′)
USP3 (NM_001025424.1)	CCTGACCATGATTCATCTAC
	GTTACTACCACTGACCAAAC
SIRT3 (NM_001106313.2)	CAGTATGACATCCCGTACCC
	TGTGTAGAGCCGCAGAAG
GAPDH (NM_017008.4)	GGAGTCTACTGGCGTCTTCAC
	ATGAGCCCTTCCACGATGC

### Immunoblotting and co-immunoprecipitation assays

Proteins were extracted by adding Immunoprecipitation (IP) Lysis Buffer (20 mM Tris-HCl, pH 7.6; 150 mM NaCl; 1 mM EDTA; 0.5% NP-40; 10% glycerol; 1 mM PMSF; protease inhibitor cocktail), incubated with anti-USP3 (ab80597, Abcam) and anti-SIRT3 antibodies (#5490, CST), and followed by overnight incubation with Protein A/G beads. Rabbit immunoglobulin G (IgG) (Santa Cruz Biotechnology, USA) was used as a control. The beads were washed, re-suspended in a loading buffer, and boiled.

For immunoblotting, proteins were isolated using RIPA lysis solution (Thermo Fisher Scientific, China) and resolved following the addition of SDS-PAGE. The proteins were subsequently transferred to a polyvinylidene fluoride membrane, sealed in 5% skimmed milk, and incubated with optimally diluted primary and secondary antibodies sequentially. After development with ECL luminous fluid (WBKLS0100, Millipore) for 5 min, protein bands were assessed using an imager (Bio-Rad, China)[31]. Antibody information was as follows: anti-USP3 (1:500, Ab82935), anti-SIRT2 (1:1 000, Ab19388), anti-SIRT3 (1:500, Ab189860), anti-SIRT6 (1:5 000, Ab191385), anti-P21 (1:1 000, Ab109199), anti-FOXO3 (1:3 000, Ab17026), anti-Ac-FOXO3 (1:500, Ab47285), anti-catalase (1:1 000, Ab52477), and anti-MnSOD (1:3 000, Ab13533). All were purchased from Abcam. Anti-SIRT1 (1:1 000, 13,161-1-AP) and anti-P16 (1:600, 10,883-1-AP) were purchased from Proteintech. Anti-Cyclin E1 (1:1 000, #20,808), anti-CDK2 (1:1 000, #2546), and anti-β-actin (1:1 000, #4970) were purchased from CST.

### ROS detection

ROS levels were detected using an Active Oxygen Detection Kit (S0033, Beyotime, China). Cells were treated, collected, and re-suspended supplemented with 1 mL of cooled PBS containing 10 µM DCFH-DA at 37°C in the dark for 20 min. The cells were tossed and mixed with the solution every 5 min. Following three cycles of washing with serum-free medium, ROS levels were determined using a flow cytometer (BD, Franklin Lakes, NJ)[32].

### Cell cycle profile analysis

Forty-eight hours after treatment, cells were fixed in 70% ice-cold ethanol and then incubated with ribonuclease A (0.1 mg/mL, Sigma) and propidium (PI) (0.05 mg/mL, Sigma) at 25°C for 30 min. DNA content was analyzed using the BD flow cytometer (Franklin Lakes, NJ)[33].

### Senescence-associated β-galactosidase (SA-β-gal) assay

Chondrocytes were stained using a Senescence β-gal Staining Kit (C0602, Beyotime, China) following the manufacturer’s instructions. Briefly, the cells were initially fixed and incubated with mixed staining solution in a CO_2_-free dry incubator at 37°C for 12 h. Subsequently, positive (blue) cell ratios of five random fields were counted in all assays using a 100× magnification bright-field microscope [34]. For each group, three pictures were taken and the positive cells dyed blue were quantified by Image J software (National Institutes of Health, Bethesda, MD).

### Statistical analysis

All data were analyzed using Prism 7.0 (GraphPad, San Diego, CA) and presented as mean ± standard deviation. Student’s t-tests or one-way analysis of variance were performed for comparison using Tukey’s post-hoc tests. *P* values less than 0.05 were considered statistical significance.

## Results

Multiple methods and technologies were performed to investigate the role of USP3 in IL-1β-induced senescence of isolated articular chondrocytes. The gene and protein expressions were detected using quantitative polymerase chain reaction and immunoblotting when USP3 was overexpressed or silenced by lentivirus in chondrocytes. The cell cycle profile was analyzed using a flow cytometer. Reactive oxygen species (ROS) levels were measured. The overexpression of USP3 was expected to hinder IL-1β-mediated cell cycle arrest, ROS generation, and chondrocyte senescence. The underlying mechanism and vital role of USP3 in chondrocyte senescence were explored.

### Overexpression of USP3 attenuates IL-1β-mediated cell senescence

**Figure 1.**
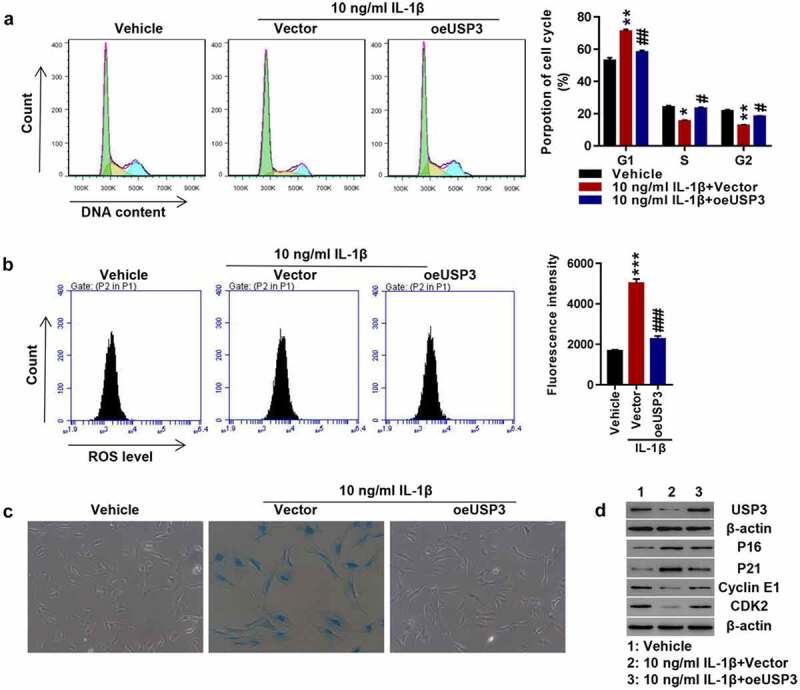
Overexpression of USP3 attenuated IL-1β-mediated chondrocyte senescence.

To investigate whether USP3 could affect chondrocyte senescence, USP3 was overexpressed in rat primary chondrocytes using lentivirus. Transfected cells were treated with 10 ng/mL recombinant IL-1β protein. The results revealed that IL-1β markedly arrested the progression of cell cycles at the G1 phase, which was potently abolished by USP3 overexpression (Figure 1a). In addition, the overexpression of USP3 abolished IL-1β-induced ROS production (Figure 1b) and cell senescence (Figure 1c). Western blotting indicated that IL-1β largely decreased USP3, Cyclin E1, and CDK2 while increased p16 and p21, and all were blocked following overexpression of USP3 (Figure 1d). The results indicated that overexpressing USP3 attenuated IL-1β-caused chondrocyte senescence.

The primary chondrocytes were transfected with USP3 over expression lentivirus or control vector virus (Vector) in the presence of recombinant IL-1β protein or vehicle. (**A**) Cell cycle detection using flow cytometry. (**B**) ROS level detection using flow cytometry. (**C**) Cell senescence shown by SA-β-Ga1 staining. (**D**) Levels of USP3, p16, p21, Cyclin E1, and CDK2 detected by Western blotting. Each data shows mean ± standard deviation from three independent biological experiments. Compared with vehicle, *p < 0.05; **p < 0.01; ***p < 0.001. And compared with IL-1β + vector, #*p* < 0.05; ##*p* < 0.01; ###*p* < 0.001.

### USP3 regulates SIRT3 expression by inhibiting SIRT3 ubiquitination

**Figure 2.**
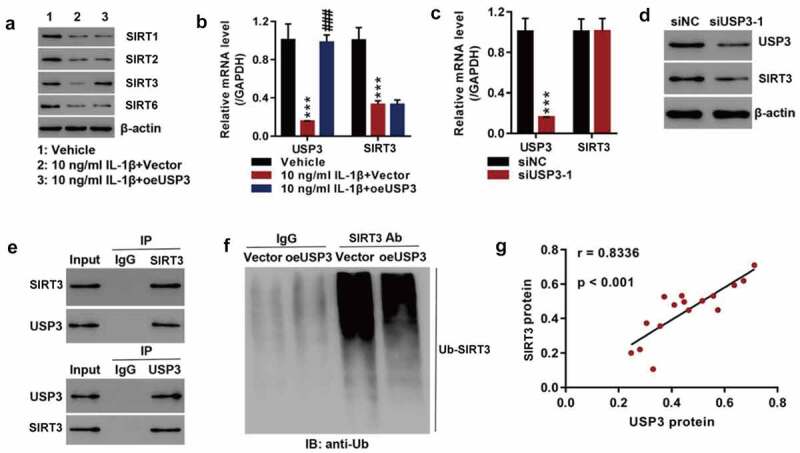
USP3 regulated SIRT3 expression probably by inhibiting SIRT3 ubiquitination.

As SIRT1, SIRT2, SIRT3, and SIRT6 are family members of SIRT, we detected the expressions of the mentioned anti-aging members and found that recombinant IL-1β proteins significantly inhibited SIRT1, SIRT2, SIRT3, and SIRT6 protein expressions. USP3 overexpression significantly increased SIRT3 at the protein level (Figure 2a) but it did not affect SIRT3 mRNA levels (Figure 2b). Silencing USP3 significantly reduced SIRT3 proteins but did not have a significant effect on SIRT3 mRNA (Figure 2c**–**2d). The findings indicated that USP3 regulated SIRT3 at the protein level. Co-IP showed that USP3 interacted with SIRT3 (Figure 2e), and the overexpression of USP3 inhibited SIRT3 ubiquitination (figure 2f). This suggested that USP3 alleviated IL-1β-induced senescence of rat chondrocytes by inhibiting SIRT3 ubiquitination, which was consistent with the finding that USP3 expression was positively correlated with SIRT3 expression in OA cartilage tissue (Figure 2g).

The primary chondrocytes were transfected with USP3 over expression lentivirus or control vector virus (Vector) in the presence of recombinant IL-1β protein or vehicle. (**A**) Levels of SIRT1, SIRT2, SIRT3, and SIRT6 were detected using Western blotting. (**B**) mRNA levels of USP3 and SIRT3 were measured using qPCR. (**C**) mRNA levels of USP3 and SIRT3 were detected using qPCR after silencing of USP3. (**D**) Protein levels of USP3 and SIRT3 were determined by Western blotting after silencing of USP3. (**E**) Co-IP assay of the interaction between USP3 and SIRT3. (**F**) SIRT3 ubiquitination assay. (**G**) Correlation analysis between USP3 and SIRT3 in 16 OA cartilage samples (r = 0.8336, P < 0.001). Each data shows mean ± standard deviation from three independent biological experiments. ***p < 0.001 vs vehicle or siNC; ###*p* < 0.001 vs. IL-1β + vector.

### Inhibition of SIRT3 suppresses the effect of USP3 overexpression in cell senescence

**Figure 3.**
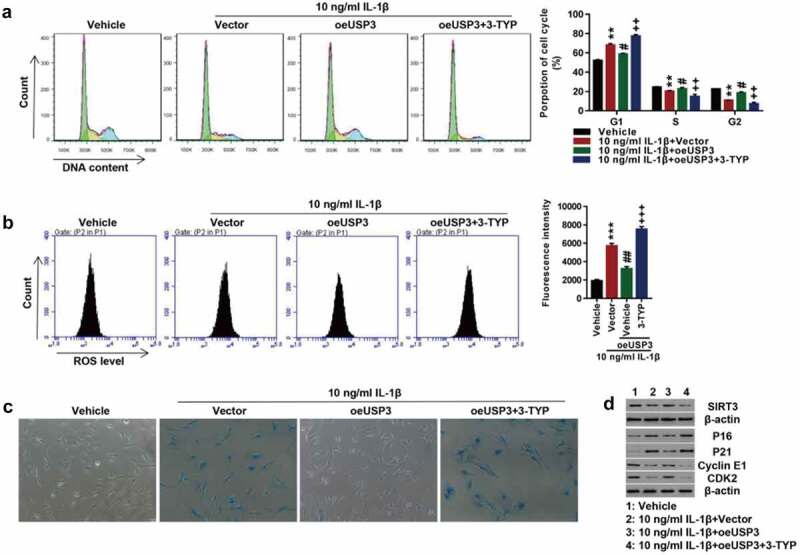
Inhibition of SIRT3 suppressed the effect of USP3 overexpression on IL-1β-mediated cell senescence.

To investigate the role of SIRT3 in IL-1β-induced chondrocyte senescence, the USP3-overexpressing cells were treated with IL-1β protein along with SIRT3 inhibitor 3-TYP. USP3 overexpression abolished IL-1β-induced cell cycle arrest. The inhibition of SIRT3 blocked the effects of USP3 overexpression on cell cycles (Figure 3a). Furthermore, the inhibition of SIRT3 abolished the suppressive effect of USP3 overexpression on IL-1β-induced ROS production (Figure 3b) and cell senescence (Figure 3c). The administration of SIRT3 inhibitor 3-TYP reversed the effects of USP3 on IL-1β-induced decrease in USP3, Cyclin E1, and CDK2 and increase in p16 and p21 (Figure 3d). These data suggested that SIRT3 inhibition suppressed the protection of USP3 overexpression on IL-1β-induced chondrocyte senescence.

Rat primary chondrocytes following USP3 overexpression were treated with IL-1β protein (10 ng/mL) along with SIRT3 inhibitor 3-TYP (50 μM). (**A**) Cell cycle detection by flow cytometry. (**B**) ROS levels by flow cytometry. (**C**) Cell senescence shown by SA-β-Ga1 staining. (**D**) Expressions of USP3, p21, p16, Cyclin E1, and CDK2 detection using Western blotting. Each data shows mean ± standard deviation from three independent biological experiments. **p < 0.01; ***p < 0.001 compared with vehicle; #*p* < 0.05; ##*p* < 0.01 compared with IL-1β + vector; ++*p* < 0.01; +++*p* < 0.001 compared with IL-1β + vector.

### Overexpression of SIRT3 attenuates USP3 interfere-induced cell senescence

**Figure 4.**
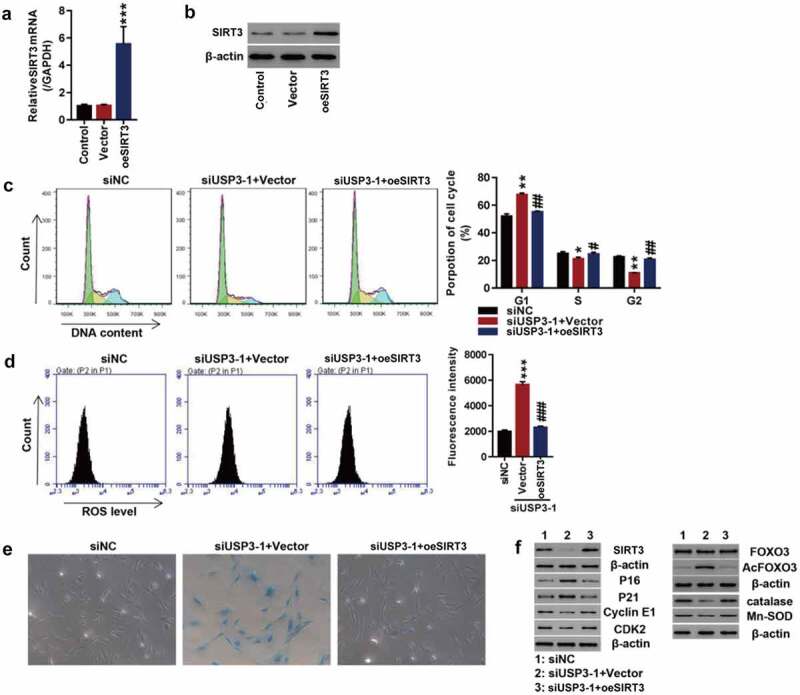
Overexpression of SIRT3 attenuated USP interfere-induced cell senescence.

To further investigate SIRT3 action on IL-1β-induced senescence, SIRT3 was successfully overexpressed at both mRNA and protein levels in primary chondrocytes of the rats (Figure 4a-b). Efficiency of USP3-silencing was shown in Figure 2c**-D**. Data showed that USP3 silencing significantly arrested cell cycle progression at phase G1, abolished following SIRT3 overexpression (Figure 4c). Furthermore, SIRT3 overexpression abolished USP3-silencing-induced ROS production (Figure 4d) and cell senescence (Figure 4e). In addition, USP3 silencing significantly decreased SIRT3, Cyclin E1, CDK2, catalase, and Mn-SOD and increased p16, p21, and Ac-FOXO3, which were blocked by SIRT3 overexpression (figure 4f). The results indicated that the overexpression of SIRT3 attenuated USP interfere-induced cell senescence.

SIRT3 was overexpressed in USP3-silencing rat primary chondrocytes. (**A–B**) Confirmation of SIRT3 overexpression using qPCR (**A**) and Western blotting (**B**) ***p < 0.001 vs. vector. (**C**) Cell cycle detection by flow cytometry. (**D**) ROS levels measured by flow cytometry. (**E**) Cell senescence shown by SA-β-Ga1 staining. (**F**) Expressions of SIRT3, p16, p21, Cyclin E1, CDK2, FOXO3, Ac-FOXO3, catalase, and Mn-SOD detected using Western blotting. Each data shows mean ± standard deviation from three independent biological experiments. *p < 0.05; **p < 0.01; ***p < 0.001 compared with siNC; #*p* < 0.05; ##*p* < 0.01; ###*p* < 0.001 compared with siUSP3-1 + vector.

### Overexpression of SIRT3 attenuates chondrocyte senescence probably by deacetylating FOXO3

**Figure 5.**
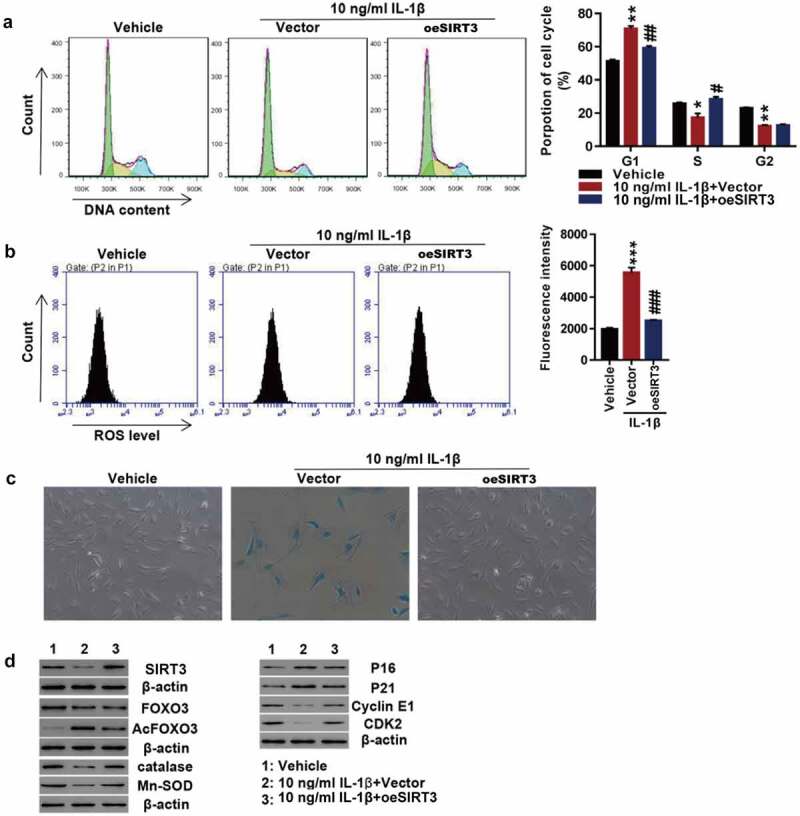
Overexpression of SIRT3 attenuated cell senescence probably by deacetylating FOXO3.

To study how SIRT3 regulates cell senescence, IL-1β was used to treat rat SIRT3-overexpressing primary chondrocytes. The overexpression of SIRT3 potently reversed IL-1β recombinant protein-arrested cell cycle progression (Figure 5a). In addition, IL-1β-induced ROS production (Figure 5b) and cell senescence (Figure 5c) were blocked by SIRT3 overexpression. Furthermore, IL-1β treatment caused significant downregulation of SIRT3, FOXO3, catalase, Mn-SOD, Cyclin E1, and CDK2 and upregulation of Ac-FOXO3, p16, and p21, which were blocked by SIRT3 overexpression (Figure 5d).

The primary chondrocytes were transfected with SIRT3 over expression lentivirus or control vector virus (Vector) in the presence of recombinant IL-1β protein or vehicle. (**A**) Cell cycle detection by flow cytometry. (**B**) ROS levels measured by flow cytometry. (**C**) Cell senescence shown by SA-β-Ga1 staining. (**D**) Levels of SIRT3, FOXO3, Ac-FOXO3, catalase, Mn-SOD, p16, p21, Cyclin E1, and CDK2 detected by Western blotting. Each data shows mean ± standard deviation from three independent biological experiments. *p < 0.05; **p < 0.01; ***p < 0.001 compared with vehicle; #*p* < 0.05; ##*p* < 0.01; ###*p* < 0.001 compared with IL-1β + vector.

## Discussion

It has been reported that IL-1β has involved in the occurrence and progression of OA[35]. Studies have highlighted that this inflammatory cytokine was significantly increased in both chondrocytes and synovial cells from patients with OA [36,37]. Chondrocytes, expressing the receptor of IL-1β, are the major cellular targets for this cytokine. Therefore, IL-1β was chosen for chondrocyte treatment in this study. IL-1β processed chondrocytes could arrest the progression of cell cycles in the G1 phase, induce ROS production, and promote chondrocyte senescence. These findings have further confirmed that IL-1β plays a central role in OA.

As one of the five types of deubiquitinases (DUBs), USP3 trims ubiquitin chains from substrate-distal ends and rescues proteins from being degraded[38]. USPs have been implicated in various pathological processes including rheumatoid arthritis and autophagy [38,39]. Ogrunc et al. have shown that USP1 regulated cellular senescence by controlling genomic integrity[40]. Fukuura et al. have shown that USP17 prevented cell senescence by stabilizing SET domain-containing protein 8 and transcriptionally repressing p21[41]. More importantly, we have shown that USP3 deubiquitinates TRAF6 to mediate IL-1β-induced chondrocyte apoptosis[29]. Here, we further showed that USP3 increased SIRT3 expression levels by inhibiting SIRT3 ubiquitination and rescuing SIRT3 from degradation. Furthermore, inhibition of SIRT3 suppressed the protective effect of USP3 overexpression on IL-1β-induced senescence, and the overexpression of SIRT3 attenuated USP interfere-induced cell senescence. These findings indicate that USP3 upregulates SIRT3 levels to suppress chondrocyte senescence. The result is in agreement with a previous finding revealing that SIRT3 deficiency has been implicated in OA development, and SIRT3 activation suppresses chondrocyte degeneration[31].

SIRT3 serves as an NAD+-dependent protein deacetylase, deacetylates FOXO3, and regulates expressions of various genes being responsible for survival, apoptosis, autophagy, and metabolism[27]. The present study suggested that overexpression of SIRT3 attenuated IL-1β-induced chondrocyte senescence by deacetylating FOXO3. A study has shown that SIRT3 suppresses hyperglycemia-induced senescence of human diploid fibroblasts by deacetylating FOXO1[25]. Akasaki et al. have reported that FOXO3 is significantly decreased with joint aging[42]. Another study has indicated that overexpression of FOXO1 in chondrocytes decreases inflammatory cytokines and abolishes IL-1β effects[43]. Despite numerous studies have reported SIRT and FOXO proteins, studies on SIRT3/FOXO3 signaling in pathological processes are relatively scarce. The role of the SIRT3/FOXO3 axis in the senescence of chondrocytes is not fully understood. This study initiated experiments to demonstrate that overexpression of SIRT3 reduced the level of Ac-FOXO3 and attenuated IL-1β-induced chondrocyte senescence using various molecular biology techniques.

## Conclusions

Taken together, the present research revealed that USP3 served as a deubiquitinating enzyme to upregulate the expression of SIRT3. The upregulation of SIRT3 promoted FOXO3 deacetylation to inhibit IL-1β-mediated ROS production and cell senescence. The findings indicated a pivotal role of USP3/SIRT3/FOXO3 in OA, allowing for a better understanding of regulating chondrocyte senescence in OA. Although further investigation of the underlying mechanisms is necessary, the current study provides us with a feasible therapeutic approach for OA.

## Data Availability

The data that support the findings of this study are available from the corresponding author upon reasonable request.

## Highlights

1. Overexpression of USP3 inhibits IL-1β-mediated cell cycle arrest, ROS generation, and chondrocyte senescence.
2. SIRT3 inhibition blocks the protective effect of USP3 on cell senescence.

SIRT3 attenuates cell senescence by deacetylating FOXO3 in IL-1β-mediated chondrocyte senescence
